# The relationship of primary health care use with persistence of insomnia: a prospective cohort study

**DOI:** 10.1186/1471-2296-13-8

**Published:** 2012-02-16

**Authors:** Richard A Hayward, Kelvin P Jordan, Peter Croft

**Affiliations:** 1 Arthritis Research UK Primary Care Centre, Keele University, Keele, Staffs ST5 5BG, UK; 2 Arthritis Research UK Primary Care Centre, Keele University, Keele, Staffs ST5 5BG, UK

**Keywords:** Primary health care, Insomnia, Persistence, Cohort study

## Abstract

**Background:**

Prevalence of insomnia symptoms in the general population is high. Insomnia is linked with high health care use and within primary care there are a number of treatment options available. The objective of this study was to determine the association of persistence and remission of insomnia with primary health care using a longitudinal study.

**Methods:**

A postal survey of registered adult (over 18 years) populations of five UK general practices, repeated after 1 year, linked to primary care records. Baseline survey responders were assessed for persistence of insomnia symptoms at 12 months. The association of primary care consultation or prescription for any mood disorder (defined as anxiety, depression, stress, neurosis, or insomnia) in the 12 months between baseline and follow-up surveys with persistence of insomnia was determined.

**Results:**

474 participants reporting insomnia symptoms at baseline were followed up at 12 months. 131(28%) consulted for mood problem(s) or received a relevant prescription. Of these 100 (76%) still had insomnia symptoms at one year, compared with 227 (66%) of those with no contact with primary care for this condition (OR 1.37; 95% CI 0.83, 2.27). Prescription of hypnotics showed some evidence of association with persistence of insomnia at follow-up (OR 3.18; 95% CI 0.93, 10.92).

**Conclusion:**

Insomniacs continue to have problems regardless of whether or not they have consulted their primary care clinician or received a prescription for medication over the year. Hypnotics may be associated with persistence of insomnia. Further research is needed to determine more effective methods of identifying and managing insomnia in primary care. There may however be a group who have unmet need such as depression who would benefit from seeking primary health care.

## Funding

This work was supported by Arthritis Research UK Primary Care Centre and the Department of Medicines Management, Keele University. Richard A Hayward is receiving an In-Practice Fellowship from the National Institute for Health Research

## Background

The reported prevalence of insomnia in the general population is high, varying between 6% and 37% depending on the criteria used. Insomnia may be defined as simply self-reported "difficulty sleeping" on one or more nights in the previous month or more stringently using, for example, the DSM-IV criteria of primary insomnia which excludes insomnia associated with psychiatric or physical illness [1-8]. Insomnia is a common accompaniment to anxiety and depression, with around two-thirds of those with insomnia reporting anxiety or depression and it may be an early symptom of depression [6-10]. This link with psychological distress may be one reason for the high health care use in people with insomnia, an increased number of accidents at work, presence of comorbidity and greater use of medication than good sleepers [7,11,12].

Most studies of insomnia are cross-sectional, hence the natural history of insomnia is not fully explored. There are several studies which have examined the outcome in primary care for people presenting with insomnia, and in particular how this relates to management. Foley et al. observed a persistence of insomnia symptoms of 52% in elderly patients in the community over a three year period, persistence being significantly and independently associated with physical disability, depression and sedative use [13]. Janson et al. in a 10 year prospective study of adult men in the community reported associations of insomnia with medical and psychiatric disorders, remission being related to weight loss and smoking cessation; however medication use was not reported [4]. Simon et al. in a community study with a 3 month telephone follow-up likewise found a positive association between insomnia and healthcare use, although this appears to have been closely linked to depression [14].

There are a plethora of treatments available to primary care clinicians for the management of patients with insomnia, both pharmacological and non-pharmacological. This may bear testament to the fact that no treatment is both effective and without side effects, although cognitive behavioural therapy and the melatonin agonist ramelteon appear promising [7,8,15-18]. Improvements in insomnia following CBT interventions delivered over the internet persisted at 6 month follow-up [18].

We previously reported results from a baseline health survey, linked to medical record review, of health care use for insomnia and mood problems [9]. Of those reporting insomnia at baseline, 30% had either a consultation for a mood disorder or a prescription during the following 12 months, with 21% receiving antidepressants and 8% receiving hypnotics.

This study aimed to use a 12 month follow-up survey of this population to determine the relationship of prescribed medication and consultations for insomnia or anxiety and depression with persistence of self-reported insomnia symptoms.

## Methods

This was a prospective cohort study linking baseline and 12 month survey to medical record review [1]. Ethical approval for the study was granted by the North Staffordshire Local Research Ethics Committee. Random selection of patients aged 18 plus was made by computer from the registered populations of five general practices in the UK. More than 95% of the UK population are registered with a general practice, which provides a convenient sampling frame of the general population in a locality. One thousand patients from each practice (a total of 5000 patients) were selected. 115 people were excluded as they were currently in hospital, recently died, left the practice, or were excluded by their GP as suffering from severe mental illness. 4885 questionnaires were therefore sent out in April 2000 with a follow-up questionnaire 1 year later. This study was carried out to investigate headaches and general health, but included questions about sleep, which we have used as a basis for this new investigation.

Jenkins et al. have developed four questions related to insomnia and these were included in both baseline and one year follow-up questionnaires [19]. These were:- "Over the last month did you i) Have trouble falling asleep, ii) Wake up several times a night, iii) Have trouble staying asleep, iv) Wake up after your usual amount of sleep feeling tired and worn out'?". Possible responses were 'Not at all, On some nights, On most nights'. Our main definition of insomnia was based on answering one or more of these questions"on most nights".

The Hospital Anxiety and Depression Scale (HADS) was used to assess anxiety and depression [20,21]. Respondents who scored 8 or over on either of the two domains were classified as having self-reported anxiety or depression.

Pain is closely associated with insomnia, and is likely to be a confounder between poor sleep and the use of primary health care [22]. The participants were asked to shade in on a diagram of a blank body manikin any aches or pains lasting for 1 day or more in the previous month. Number of pain sites was categorised into 0, 1-3 and 4-7 sites.

Consent to examine their medical records was sought from people in the survey population. For the 1 year between the baseline and follow-up questionnaires, relevant consultations and prescription data for mood disorders were extracted. These were defined as either insomnia, or anxiety or depression recorded as the presenting problem, or prescriptions in the groups listed below. Prescription medication data was extracted from the records by British National Formulary categories (BNF) 4.1 and 4.3. All antidepressants (BNF 4.3), including tricyclics and serotonin reuptake inhibitors may be used in the treatment of insomnia, whereas hypnotics (BNF 4.1.1) and anxiolytics (BNF 4.1.2) have different clinical applications. Anxiolytics such as diazepam, chlordiazepoxide and lorazepam were little used in the treatment of insomnia in this group. Categories 4.1.3 (barbiturates) and 4.3.2 (monoamine oxidase inhibitors) had not been prescribed to these patients. The hypnotics (BNF 4.1.1), include the benzodiazepine receptor agonists zopiclone, zolpidem, zaleplon, shorter acting benzodiazepines, temazepam and lometazepam and the longer acting nitrazepam and flurazepam.

### Analysis

Respondents to the baseline questionnaire who answered positively to any of the insomnia questions on "most nights" were investigated for their use of healthcare over the next year and analysed as regards to their responses to the insomnia questions in the follow-up questionnaire.

The association of any self-reported insomnia symptom at follow-up with relevant health care use for mood disorder was determined after adjustment for self reported baseline anxiety and depression, age, gender, number of insomnia symptoms and number of pain areas. The analysis was repeated for the four individual insomnia symptoms. Analysis was performed using logistic regression with results presented as odds ratios and 95% confidence intervals. SPSS 15.0 for Windows was used.

## Results

**Figure 1:**
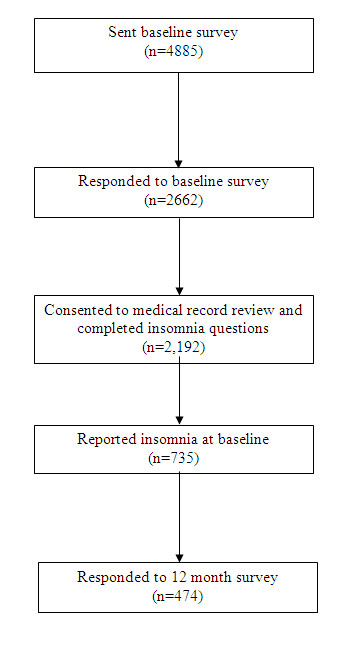
**Flow diagram of participants**.

A flow diagram of participants is given in Figure 1. Of the 4885 questionnaires sent out, 2662 were returned at baseline. Following adjustment for deaths and change of address the response rate was 56%. Response was higher in females (61% compared to 51% in males) and in older age groups (65% in those aged 65+ compared to 43% in those aged 18-35). 2363 answered all the sleep questions and of these 2192 consented to examination of their medical records. Of the 2192 responders, 735 reported an insomnia symptom at baseline. Of these, 474 (64%) consented to follow-up at 12 months, returned a follow-up questionnaire and completed the follow-up insomnia questions.

Compared to those reporting insomnia at baseline who did not consent to record review or did not complete the questionnaire at follow-up, the 474 participants who were followed up were no different in terms of gender (female: 63% v. 60%), age (mean 52 v. 51), levels of anxiety or depression at baseline (68% v. 70%), or reporting multiple pains at baseline (pain in more than 4 sites: 52% v. 47%). When further compared with those who reported insomnia at baseline and consented to record review but did not complete the follow-up questionnaire, in the 12 months after baseline the 474 participants had a similar prevalence of consultation for a mood disorder (18% v. 20%) and hypnotic prescription (8% v. 8%), but slightly lower level in terms of anti-depressant prescription (19% v. 23%).

### Persistence of any insomnia symptom

327 (69%) of the 474 participants continued to suffer from an insomnia symptom at follow-up. Of the 343 who had no consultation or prescription for a mood disorder during the one year follow-up period, 227 (66%) still had insomnia at 12 months. Of the remaining 131 who had consulted or been given a prescription for a mood disorder, 100 (76%) still had insomnia at 12 months. In the unadjusted analysis, any consultation or prescription for mood disorder was associated with persistence of insomnia problems (OR 1.65; 95% CI 1.04, 2.61) although this association was reduced after adjustment for age, gender, pain, anxiety or depression, and number of symptoms (OR 1.37; 95% CI 0.83, 2.27). Baseline anxiety and depression had a similar level of association as consultation or prescription had with persistence of insomnia problems (OR 1.39; 95% CI 0.87, 2.22).

Prescriptions for hypnotics were strongly associated with persistence of insomnia in the unadjusted analysis with 33 (92%) of those receiving a hypnotic still having insomnia at 12 months (OR 5.38; 95% CI 1.63,17.86). This association was still strong although not statistically significant after adjustment (OR 3.18; 95% CI 0.93, 10.92). There was no association of persistent insomnia with antidepressants. (Table 1)

**Table 1:** Reporting of insomnia at 12 months by health care use in those who self-reported insomnia at baseline

		Reported Insomnia 12 months			
	**Total**	**Yes**	**No**	**OR^a^**	**OR^b^**
		***n *(%)**	***n***		

Hypnotics					
No	438	294 (67)	144	1.00	1.00
Yes	36	33 (92)	3	5.38 (1.63, 17.86)	3.18 (0.93, 10.92)

Any antidepressant					
No	383	260 (68)	123	1.00	1.00
Yes	91	67 (74)	24	1.32 (0.79, 2.21)	1.12 (0.64, 1.96)

Any mood					
consultation No	343	227 (66)	116	1.00	1.00
or prescription Yes	131	100 (76)	31	1.65 (1.04, 2.61)	1.37 (0.83, 2.27)

^a ^unadjusted

^b ^adjusted for age, gender, number of insomnia symptoms, pain, anxiety or depression at baseline

### Persistence of individual symptoms

In those reporting "trouble falling asleep" at baseline, having a consultation or prescription for a mood disorder was suggestive of higher rates of persistence of this symptom at 12 months although the association was not statistically significant (adjusted OR 1.98; 95% CI 0.98, 4.02). A similar finding occurred for the symptom "trouble staying asleep" (adjusted OR 1.78; 95% CI 0.93, 3.43). There was little apparent relationship of persistence with health care use for either of the other two symptoms: waking up several times or waking up tired and worn out. (Table 2)

**Table 2:** Reporting of individual insomnia symptoms at 12 months by health care use in those who self-reported insomnia at baseline

			Reported Insomnia 12 months			
		**Total**	**Yes**	**No**	**OR^a^**	**OR^b^**
			***n *(%)**	***n***		

Trouble falling asleep	Any mood consultation No or prescription Yes	97 66	51 (53) 46 (70)	46 20	1.00 2.08 (1.07, 4.01)	1.00 1.98 (0.98, 4.02)

Wake up several times	Any mood consultation No or prescription Yes	269 110	177 (66) 70 (64)	92 40	1.00 0.91 (0.57, 1.44)	1.00 0.78 (0.46, 1.32)

Trouble staying asleep	Any mood consultation No or prescription Yes	167 74	92 (55) 53 (72)	75 21	1.00 2.06 (1.14, 3.71)	1.00 1.78 (0.93, 3.43)

Wake up tired and worn out	Any mood consultation No or prescription Yes	177 89	93 (53) 55 (62)	84 34	1.00 1.46 (0.87, 2.46)	1.00 1.36 (0.77, 2.42)

^a ^unadjusted

^b ^adjusted for age, gender, pain, number of insomnia symptoms, anxiety and depression at baseline

## Discussion

This study linked baseline and 12 month postal surveys in a general population sample with primary care medical records. Those receiving primary care for mood disorders did not have better outcomes in terms of remission of their insomnia symptoms. In those reporting insomnia at baseline, three-quarters of those receiving health care management still had symptoms at follow-up.

This study found persistent insomnia symptoms were higher in people prescribed hypnotic drugs, but there was no association with antidepressant use. Foley et al. also found a close relationship between sedative medication and persistence of insomnia symptoms over 3 years in an elderly population in the community [13]. In elderly women (aged 70-75 yrs) Byles et al. found that over 75% of those taking hypnotics at baseline continued to take these drugs at follow-up 4 years later [23] Belleville et al. found that withdrawal of hypnotics over an 8 week period in a group of chronic users of this medication resulted in over 68% of participants in the study being drug free, and 51% still being drug free at 6 months. This was independent of whether or not the tapering dose of hypnotics was combined with self-help CBT in the form of a graded program of literature [24]. Tom et al. in their cohort of middle aged women studied over six years found that persistence of insomnia was related both to psychiatric and physical symptoms and a past history of insomnia [25].

Regardless of primary care use, two-thirds of this sample still had insomnia symptoms at follow-up. This suggests that in most people with insomnia symptoms in the community these symptoms are chronic. The majority did not consult or have a prescription for this problem, some possibly choosing to self medicate. Our previous work showed a clear link between insomnia and depression, this non-consulting group may have unmet need such as depression [9]. Alternatively they may see that primary care is routinely only offering drug treatments with possible side effects. Horne, for example, suggests that some people perceive medication negatively as unnatural or dangerous and prefer to self-medicate [26]. Included in this group will also be some with milder symptoms.

Remission is seen in one-third of people with insomnia at baseline who do not have a consultation or prescription. This no doubt represents the temporary nature of the insomnia stimulus in some but is broadly in agreement with Foley et al. who found a 48% remission of insomnia after 3 years in the community on no sedative medication, decreasing to 33-37% at follow-up when sedative medication was taken [13]. Janson et al. found a remission rate of 65% in men aged 30-69 yrs after a 10 year follow-up, associated with smoking cessation and weight loss, although medication use was not recorded [4]. Even in the more severe insomniacs found in a sleep medicine clinic in the US, Rosenthal et al. (2008), reported the remission of insomnia over 3 to 5 years to be 26% [27]. In Morin et al's general population study of those complaining of poor sleep, 31% had what was described as insomnia syndrome, which included those who were dissatisfied with sleep and having insomnia for 3 nights a week for at least a month or those who took hypnotics. At 3 year follow-up 30% of the insomnia syndrome group had persistence of their symptoms and only 8% of this group had spontaneous remission [28].

Hypnotic use appears associated with an increased likelihood of persistence. There are several interpretations of this result. Hypnotic medication may be ineffective in the long-term in primary care and potentially exacerbates the problem, this exacerbation may be due to a specific problem of tolerance and addiction related to both persistence and medication use [29]. The group of people with insomnia who receive prescriptions for hypnotics may in some way be "different" to people who do not take hypnotics or do not consult primary care, in ways which might explain poor outcome despite medication.

Whatever the explanation, however, primary care is having little impact on community levels of chronic insomnia with its current range or style of medications. However cognitive behavioural therapy (CBT) may be as effective and with less side effects than medication [30]. Internet-based CBT may be attractive in that it is a low-cost alternative to face to face CBT [18]. The GP may wish first to give sleep hygiene advice or organise CBT if this is available when faced with a patient with complaints of insomnia.

A strength of this study is its longitudinal design with surveys at baseline and 12 months linked by consultation and prescription data. Associations were adjusted for age, gender, and number of pain areas, all of which have been shown to be related to both insomnia and health care use [12]. Existence of other chronic illnesses, for example cardiac, respiratory disease or diabetes, were not measured and may be associated with insomnia and hence may have an influence on the results [31]. We did not measure over the counter medication which may have been used to self-treat insomnia. Response at baseline was 56% and there were some age and gender differences in response. As with all cohort studies, there was further attrition at follow-up. However, there were few differences between those completing follow-up questionnaires and those who did not in demographics, self-reported anxiety or depression, self-reported pain, or health care use for a mood disorder. Whilst any selective non-response may affect the overall estimates of rates of consultation and prescription, it is unlikely to affect the associations found.

Consultation data for mood problems and prescriptions for hypnotics and antidepressants were examined for 1 year between the questionnaires at baseline and follow-up, so it was not possible to ascertain whether health care use was new or ongoing. The study was set in North Staffordshire, UK, an area more deprived than the UK average. However data were collected from five general practices which should reduce the likelihood that health care use was influenced by unusual practice characteristics.

## Conclusions

Those with insomnia symptoms continue to have problems regardless of whether they receive primary care management or not. Hypnotic use appears to be related to persistence of insomnia. Further research is needed to determine the most effective methods of identifying and managing insomnia in primary care. There is also a large group of people with persistent insomnia who do not seek primary health care. The reasons for this need to be established as they may have unmet need such as depression and may benefit from seeking primary health care.

## Competing interests

The authors declare that they have no competing interests.

## Authors' contributions

RH led the design of the study and the drafting of the manuscript. KJ participated in the design of the study, performed the analysis and contributed to the manuscript. PC participated in study design and helped to draft the manuscript. All authors read and approved the final manuscript.

## Pre-publication history

The pre-publication history for this paper can be accessed here:

http://www.biomedcentral.com/1471-2296/13/8/prepub

## References

[B1] Morphy H Dunn KM Lewis M Boardman HF Croft PR Epidemiology of insomnia: a longitudinal study in a UK population Sleep 2007 30 274 280 17425223

[B2] Stewart R Besset A Bebbington P Brugha T Lindesay J Jenkins R Singleton N Meltzer H Insomnia comorbidity and impact and hypnotic use by age group in a national survey population age 16 to 74 years Sleep 2006 29 1391 1397 17162985 10.1093/sleep/29.11.1391

[B3] Phillips B Mannino DM Does insomnia kill? Sleep 2005 28 965 970 16218079 10.1093/sleep/28.8.965

[B4] Janson C Lindberg E Gislason T Elmasry A Boman GP Insomnia in men-a 10-year prospective population based study Sleep 2001 24 425 430 11403527 10.1093/sleep/24.4.425

[B5] Pellesen S Nordhus Nielsen H Havik OE Kvale G Johnsen BH Skjotskitt S Prevalence of insomnia in the adult Norwegian population Sleep 2001 24 771 779 11683480

[B6] Ohayon MM Epidemiology of insomnia: what we know and what we still need to learn Sleep Med Rev 2002 6 97 111 10.1053/smrv.2002.0186 12531146

[B7] Hamblin JE Insomnia: an ignored health problem Prim Care Clin Office Pract 2007 34 659 674 10.1016/j.pop.2007.05.009 17868765

[B8] Panossian LA Avidan AY Review of Sleep Disorders Med Clin N Am 2009 93 407 425 10.1016/j.mcna.2008.09.001 19272516

[B9] Hayward R Jordan KP Croft P Health care use in persons with insomnia: a longitudinal study Br J Gen Pract 2010 60 334 340 10.3399/bjgp10X501822 20423585 PMC2858531

[B10] Lustberg L Reynolds CF Depression and insomnia: questions of cause and effect: Sleep Med Rev 2000 4 253 262 10.1053/smrv.1999.0075 12531168

[B11] Leger D Guilleminault C Bader G Levy E Paillard M Medical and socio-professional impact of insomnia Sleep 2002 25 621 625 12224841

[B12] Chevalier H Los F Boichut D Bianchi M Nutt DJ Hajak G Hetta J Hoffman G Crowe C Evaluation of severe insomnia in the general population: results of a European multinational survey J Psychopharm 1999 13 Suppl 1 S21 4 10.1177/026988119901304S04 10667452

[B13] Foley DJ Monjan A Simonsick EM Wallace RB Blazer DG Incidence and remission of insomnia among elderly adults: an epidemiological study of 6,800 persons over three years Sleep 1999 22 Suppl 2 S366 72 10394609

[B14] Simon GE Von Korff M Prevalence, Burden, and Treatment of Insomnia in Primary Care Am J Psychiatry 1997 154 1417 1423 9326825 10.1176/ajp.154.10.1417

[B15] Benca RM Diagnosis and treatment of chronic insomnia: a review Psychiatric Services 2005 56 332 343 10.1176/appi.ps.56.3.332 15746509

[B16] Vincent N Lionberg C Treatment Preference and Patient Satisfaction in Chronic Insomnia Sleep 2001 24 411 417 11403525 10.1093/sleep/24.4.411

[B17] Jacobs GD Pace-Schott EF Stickgold R Otto MW Cognitive Behavioural Therapy and Pharmacotherapy for Insomnia Arch Intern Med 2004 164 1888 1896 10.1001/archinte.164.17.1888 15451764

[B18] Ritterband LM Thorndike FP Gonder-Frederick LA Magee JC Bailey ET Saylor DK Morin CM Efficacy of an internet-based behavioural intervention for adults with insomnia Arch Gen Psych 2009 66 692 698 10.1001/archgenpsychiatry.2009.66 PMC3723339 19581560

[B19] Jenkins CD Stanton B-A Niemcryk SJ Rose RM A Scale for the Estimation of Sleep Problems in Clinical Research J Clin Epidemiol 1988 41 313 321 10.1016/0895-4356(88)90138-2 3351539

[B20] Zigmond AS Snaith RP The Hospital Anxiety and Depression Score Acta Psychiatr Scand 1983 67 361 370 10.1111/j.1600-0447.1983.tb09716.x 6880820

[B21] Bjelland I Dahl AA Haug TT Neckelmann D The validity of the hospital anxiety and depression scale an updated literature review J Psychosom Res 2002 52 69 77 10.1016/S0022-3999(01)00296-3 11832252

[B22] Ohayon MM Relationship between chronic painful physical condition and insomnia J Psychiat Res 2005 39 151 159 10.1016/j.jpsychires.2004.07.001 15589563

[B23] Byles JE Mishra GD Harris MA The experience of insomnia among older women Sleep 2005 28 8 972 979 16218080 10.1093/sleep/28.8.972

[B24] Belleville G Guay C Guay B Morin C Hypnotic taper with self-help treatment of insomnia: a randomized clinical trial J of Consulting and Clin Psychol 2007 75 2 325 335 10.1037/0022-006X.75.2.325 17469890

[B25] Tom SE Kuh D Guralnik JM Mishra GD Patterns in trouble sleeping among women at mid-life: results from a British prospective cohort study J Epidemiol Community Health 2009 63 974 979 10.1136/jech.2008.079616 19608560 PMC3267631

[B26] Horne R Patient's beliefs about treatment: the hidden determinant of treatment outcome? J Psychosom Res 1999 47 491 495 10.1016/S0022-3999(99)00058-6 10661596

[B27] Rosenthal LD Dolan DC Taylor DJ Griesner E Long-term follow-up of patients with insomnia Proc (Bayl Univ Med Cent) 2008 21 264 265 10.1080/08998280.2008.11928409 PMC2446417 18628925

[B28] Morin CM Belanger L LeBlanc M Ivers H Savard J Espie C Merette C Baillargeon L Gregoire J-P The natural history of insomnia: a population based 3-year study Arch Intern Med 2009 169 447 453 10.1001/archinternmed.2008.610 19273774

[B29] National Institutes of Health Statement regarding the treatment of insomnia, National Institutes of Health State of the Science Conference Statement, Manifestations and management of chronic insomnia in adults June 13-15, 2005 Sleep 2005 28 1049 1057 16268373 10.1093/sleep/28.9.1049

[B30] Sivertsen B Omvik S Pallesen S Pallesen S Cognitive behavioural therapy vs zopiclone for treatment of chronic primary insomnia in older adults: a randomized controlled trial JAMA 2006 295 2851 2858 10.1001/jama.295.24.2851 16804151

[B31] Ohayon MM Interlacing sleep, pain, mental disorders and organic diseases J Psychiat Res 2006 40 677 679 10.1016/j.jpsychires.2006.09.005 17027596

